# Updating a conceptual model of effective symptom management in palliative care to include patient and carer perspective: a qualitative study

**DOI:** 10.1186/s12904-024-01544-x

**Published:** 2024-08-19

**Authors:** Emma J. Chapman, Carole A. Paley, Simon Pini, Lucy E. Ziegler

**Affiliations:** 1 Academic Unit of Palliative Care, Worsley Building, University of Leeds, Clarendon Way, LS2 9NL UK; 2 Division of Psychological and Social Medicine, Worsley Building, University of Leeds, Clarendon Way, LS2 9NL UK

**Keywords:** Cancer, Symptom management, Hospice, Conceptual model, Communication skills, Medicines management

## Abstract

**Background:**

A conceptual model of effective symptom management was previously developed from interviews with multidisciplinary healthcare professionals (HCP) working in English hospices. Here we aimed to answer the question; does a HCP data-derived model represent the experience of patients and carers of people with advanced cancer?

**Methods:**

Semi-structured interviews were undertaken with six patients with advanced cancer and six carers to gain an in-depth understanding of their experience of symptom management. Analysis was based on the framework method; transcription, familiarisation, coding, applying analytical framework (conceptual model), charting, interpretation. Inductive framework analysis was used to align data with themes in the existing model. A deductive approach was also used to identify new themes.

**Results:**

The experience of patients and carers aligned with key steps of engagement, decision making, partnership and delivery in the HCP-based model. The data aligned with 18 of 23 themes. These were; Role definition and boundaries, Multidisciplinary team decision making, Availability of services/staff, Clinician-Patient relationship/rapport, Patient preferences, Patient characteristics, Quality of life versus treatment need, Staff time/burden, Psychological support -informal, Appropriate understanding, expectations, acceptance and goals- patients, Appropriate understanding, expectations, acceptance and goals-HCPs, Appropriate understanding, expectations, acceptance and goals- family friends, carers, Professional, service and referral factors, Continuity of care, Multidisciplinary team working, Palliative care philosophy and culture, Physical environment and facilities, Referral process and delays. Four additional patient and carer-derived themes were identified: Carer Burden, Communication, Medicines management and COVID-19. Constructs that did not align were Experience (of staff), Training (of staff), Guidelines and evidence, Psychological support (for staff) and Formal psychological support (for patients).

**Conclusions:**

A healthcare professional-based conceptual model of effective symptom management aligned well with the experience of patients with advanced cancer and their carers. Additional domains were identified. We make four recommendations for change arising from this research. Routine appraisal and acknowledgement of carer burden, medicine management tasks and previous experience in healthcare roles; improved access to communication skills training for staff and review of patient communication needs. Further research should explore the symptom management experience of those living alone and how these people can be better supported.

**Supplementary Information:**

The online version contains supplementary material available at 10.1186/s12904-024-01544-x.

## Background

A conceptual model of effective symptom management was previously developed from qualitative data derived from interviews with healthcare professionals working in English hospices to elicit their views about the barriers and facilitators of effective symptom management [1]. The model delineated the successful symptom management experience into four steps of: engagement, decision-making, partnership and delivery. Constructs contributing to these were identified (Table 1).

**Table 1:** HCP-derived constructs contributing to effective symptom management [1]

Guidelines and evidence
Experience
Training
Role definition and boundaries
Multidisciplinary team decision making
Availability of services/staff
Clinician – patient relationship/rapport
Patient preferences
Patient characteristics
Quality of life versus treatment need
Staff time/burden
Psychological support-formal
Psychological support -informal
Psychological support -for staff
Appropriate understanding, expectations, acceptance and goals- patients
Appropriate understanding, expectations, acceptance and goals-HCPs
Appropriate understanding, expectations, acceptance and goals- family friends, carers
Professional, service and referral factors
Continuity of care
Multidisciplinary team working
Palliative care philosophy and culture
Physical environment and facilities
Referral process and delays

Our original model was based solely on Healthcare professional (HCP) input. However, the perception of professionals may vary from that of patients and carers. A recent patient and professional survey of needs assessments in an oncology inpatient unit showed discrepancies between perception of unmet needs between staff and patients [2]. For this reason, we were concerned that what was deemed important by HCP working in palliative care may not mirror the concerns and experience of patients and carers.

Here we aimed to answer the question; does an HCP data-derived model represent the experience of patients and carers of people with advanced cancer?. If necessary, the original conceptual model of effective symptom management will be updated.

## Methods

### Design

Qualitative, semi-structured interviews were chosen to gain an in-depth understanding of the experience from the perspective of a range of patients and carers. All methods were carried out in accordance with the principles of the Declaration of Helsinki. Ethical approval was granted by a UK research ethics committee ( North of Scotland [2] Research Ethics Committee (20/NS/0086)). Verbal, recorded informed consent was given using a verbal consent script (Supplementary information 1). Our original intention had been to conduct interviews face to face facilitated by a set of laminated prompt cards based upon those used in the HCP interviews. However, adaptation to telephone interviews in patient’s homes was necessary due to COVID-19 restrictions and it became apparent that the card exercise did not work well remotely. We continued interviews based on the interview schedule but without the use of prompt cards. EC is a female, non-clinical senior research fellow in palliative care. She has experience of qualitative interviews and led the development of the original HCP-based model of effective symptom management [1]. Audio recordings were transcribed verbatim by a senior academic secretary.

### Recruitment

Participants who met the inclusion criteria were identified by a research nurse at the participating hospice. Eligible patients were those who met all 5 criteria:

1. Diagnosed with advanced disease (i.e., cancer that is considered to be incurable).
2. Had been referred to the participating hospice.
3. Were 18 years of age or over.
4. Were able to speak and understand English.
5. Were able to give informed consent.

Eligible carers were people who met all 4 criteria:

1. Were the informal carer of an eligible patient (who may or may not also be participating in the study).
2. Were 18 years of age or over.
3. Were able to speak and understand English.
4. Were able to give informed consent.

Patients or carers were excluded if they:

1. Exhibited cognitive dysfunction which would impede their being able to give informed consent and take part in the study.
2. Were deemed by hospice staff to be too ill or distressed.

Access to the inpatient unit was not possible at this time due to Covid-19 restrictions. The research nurse introduced the study, provided a participant information sheet and completed a consent to contact form. The first contact with the researcher was made by telephone to confirm (or not) interest in participation and answer questions. An interview time not less than 48 h after provision of the participant information sheet, was scheduled. The researcher and the participant information sheet explained the overall aim of the RESOLVE research programme to improve health status and symptom experience for people living with advanced cancer (Supplementary information 2). The verbal consent statements made it clear that this was a conversation for research purposes only and would not have any impact on the care the patient received (Supplementary information 3). Permission was granted that the researcher may contact the clinical team at the hospice if there was a serious concern for welfare that required urgent attention. Verbal informed consent was collected, and audio recorded at the start of the interview with participants answering yes or no to each of the statements in the verbal consent script (Supplementary information 3). Participants were told that we had already interviewed HCPs about what helped or hindered effective symptom management and now we wanted to understand their perspective too.

### Data Collection

Interview topic guides (Supplementary information 4 and 5) were used. Interviews were conducted by EC over the telephone and audio recorded onto an encrypted Dictaphone. Files were downloaded onto a secure University of Leeds drive and then deleted from the Dictaphone. No video was recorded. The researcher made brief field notes directly after the interview on impression, emotion and participant backgrounds that were disclosed.

### Analysis

An Excel spreadsheet was used to facilitate data management. We explored the constructs of patient and carer experience as defined by our existing model. An inductive framework analysis was used to align data with themes in the existing conceptual model. A deductive approach was also used to identify new themes not included in the original model. Two researchers (EC and CP) independently conducted framework analysis on all transcripts. Data was then compared and discussed until a consensus data set was developed. The study is reported in accordance with Standards for Reporting Qualitative Research (SRQR) recommendations [11].

## Results

Twelve participants were interviewed in their own homes by telephone. In five interviews a family member or friend was also present, and they were interviewed as a dyad. One interview was with a carer of a patient (patient not interviewed) and one interview was with a patient alone. Interviews lasted between 21 and 45 min. Basic self-declared demographic information was collected (Table 2).

**Table 2:** Characteristics of participants

	Relationship to patient	Gender	Age range (years)	Ethnicity
**Carers** (***n*** = **6**)	Wife-5 Friend -1	Female-6 Male-0	Unknown	Unknown-4 White British-1 White Scottish-1
	Cancer type	Gender	Age range (years)	Ethnicity
**Patients **(***n*** = **6**)	Breast-1 Lung-1 Colon-1 Oesophageal-1 Prostate-2	Female-1 Male-5	55-59-1 60-64-1 65-69-2 70-74-1 75-79-1	White British-6

One person was approached by a research nurse and provided with participant information sheet. However, when they spoke with the researcher on the telephone it was clear that they had not read the participant information sheet. The individual declined for the information to be read out loud with them. Informed consent could therefore not be given and an interview was not carried out. Upon reflection, this person was keen to informally chat to the researcher but was perhaps seeking social interaction rather than research participation. All other participants completed the interview as planned.

Participant background was relevant as one carer and one patient, had experience of working in healthcare and this may have shaped their experience and understanding. Analysis was based on the framework method; transcription, familiarisation, coding, applying analytical framework (conceptual model), charting, interpretation.

### Alignment

Data aligned with 18 of 23 constructs in the professional based model (Table 3). Pseudonyms are used to protect confidentiality.

**Table 3:** Alignment of patient and carer data with constructs in the HCP-based model

Themes in healthcare professional-based conceptual model	Example extract from patient and carer interviews
Role definition and boundaries	*And there’s more acceptance I think people who are nursing and who are in the admin, and I include the admin in palliative care there is more awareness that people are genuinely suffering with a, probably a terminal illness and a lot more care is put into it, rather than just delivering a service (Olivia, carer)*
Multidisciplinary team decision making	*We have a designated nurse Sarah who talks to us and digests what we’re saying in regard to symptoms, and she often goes back to the consultant to discuss it, she often has thoughts about how we can manage it but she always underpins that by going back to the consultant. (Olivia, carer)*
Availability of services/staff	*Having Martin on the syringe driver meant that the nurses came in every day and that was quite reassuring, and they were always there to talk about various different things. (Mary, carer)*
Clinician – Patient relationship/rapport	*Well they listen to me, you know… Yeah if I’m not happy with something I’ll tell them but like I say I’ve had no qualms with them at all (Terry, patient)*
Patient preferences	*That’s why I’ve said I will battle it out as long as I can to stay in my own home until I feel that I’ve no, well yes, it is time, I can’t deal with it no more, then yes I will go. I mean I did bypass the hospital and elect to go straight to (the hospice) rather than go to the hospital. (Andrew, patient)*
Patient characteristics (including reversible causes)	*Wendy is Wendy and if she can manage it she’ll do it and I have to let her because you know, she’s her own person and though I can give out a warning and Wendy heeds that warning. I’m trying not to mollycoddle her. (Patricia, carer)*
Quality of life versus treatment need	*I think it was the antipsychotic drug to help sleep and that made me feel like a robot. I’d take the medication and I’d be asleep for 12 h, then I’d wake up groggy all day, then I’d take it again, I’d sleep for another 12 h, I’d feel like, I’m just like a robot been taken out of a box, allowed to function for a few hours and then put back in the box. That’s how it felt so I did discuss it with and (nurse) I said no, I said I’m going to have to stop this and try something else (Andrew, patient)*
Staff time/burden	*Yeah having staff enough time for me, that’s another good one and being able to talk about my thoughts feelings and worries, and I do that as well (Terry, patient)*
Psychological support -informal	*I would say a lot of it was for Martin and for me personally having somebody else to take more control and feel like we were being more supported I guess with the pain and not just muddling along, it felt like we’d been muddling along for quite a long time and not getting anywhere so that support was useful, having somebody to talk everything through and somebody on the end of the phone (Mary, carer)*
Appropriate understanding, expectations, acceptance and goals- patients	*No, no they’ve all just said, we haven’t had any advice on how to manage the fatigue have we? No they’ve kind of like said it’s normal after chemo yeah (Kathleen, carer)*
Appropriate understanding, expectations, acceptance and goals-HCPs	*The important thing is to be able to discuss it and with my knowledge of medication as well (experience as a healthcare professional himself), I mean I can discuss it in depth. (Andrew, patient)*
Appropriate understanding, expectations, acceptance and goals- family friends, carers	*the consultant when Martin was in hospital said that the medication that he had been on wasn’t working which it wasn’t really and she felt that he needed something a lot more consistent so it was good but also she decided at that point that Martin was sort of end of life and didn’t have long so therefore the syringe driver would make life easier and more comfortable for him and so I can understand why it happened and as I say at the time it was the right thing to do and it did seem to work well for Martin. (Mary, Carer)*
Professional, service and referral factors	*The decision to be admitted to (the hospice) to sort out the pain and the sickness drug, although as I say that was a difficult decision for us to agree for Anthony to go into (the hospice), an amazing decision because they are so knowledgeable (Olivia, carer)*
Continuity of care	*I think it would be incredibly useful to have continuity of staff both in the hospital and for the care outside, The fact that we have 1 person who comes once a week and who takes you know not just the readings of on a medical record but also you get the feeling that they’re checking on how you’re doing and how you’re coping (Anthony, patient)* *I think that’s a very good point that yeah continuity of people is so important as Anthony said, he’s got Fiona who is on the district team who comes to see us each Wednesday and obviously you know we don’t have to go through the whole tale again. Although I know it’s all on, it’s quite a lot of it’s on screen but I think that her seeing Anthony you know and thinking well you know well he actually looks a bit better than last week or he’s looking OK *etc. etc. *is important (Olivia, carer)*
Multidisciplinary team working	*Every time I discuss it with (the nurse) as I said within a couple of hours she’s done it and for me to increase my medication back up she’s straight on to the GP, the GP’s processed the prescription within a few hours and it’s done (Terry, patient)*
Palliative care philosophy and culture	*All my treatment’s really been handled well and I mean as I say I’m very pleased with the (the hospice) staff, especially (the nurse) that deals with me. Nothing is too much for her. She does speak to my partner as well (Andrew, patient)*
Physical environment and facilities	*They’ve give me a few bits but the stuff they sent it was no good to me they sent me a bed thing for my bed which was no good (Terry, patient)*
Referral process and delays	*The one disappointing thing with the palliative care was that on the day Anthony needed to be admitted to (the hospice), we’d start the communication sort of about 1 o’clock after he’d been to (hospital) and they said this is what, you do need to go to the hospice for them to sort your drugs out, they weren’t able to admit us that evening and so Anthony was quite ill overnight and so we had to use the district team in the middle of the night, we had to get the doctor out so I suppose that is the only little disappointing thing but I guess perhaps they didn’t have a bed you know.(Olivia, carer)*

**Fig. 1:**
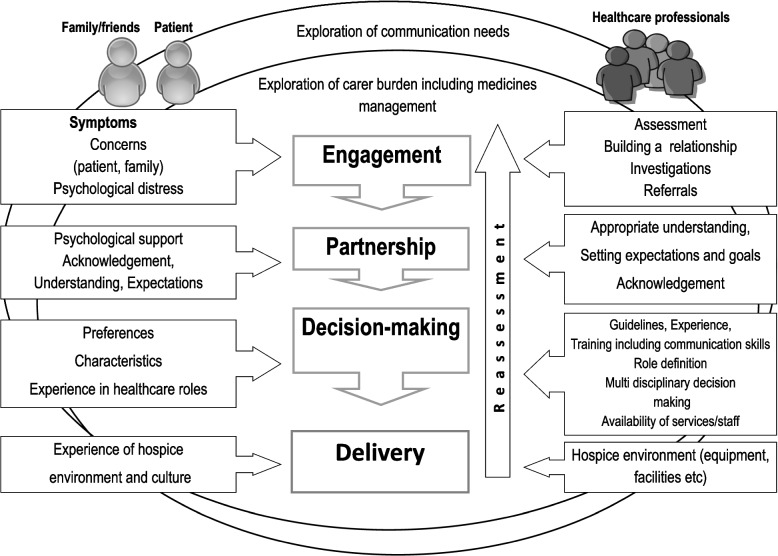
The conceptual model of effective symptom management in palliative care was updated to also reflect patient and carer perspective. Specifically, the need for support with communication and medicines management plus consideration of the carer burden were included

Four constructs that had featured in the healthcare professional based model did not feature in the patient and carer derived data. These were perhaps not unexpectedly related to characteristics of staff; Experience (of staff), Training (of staff), Psychological support (for staff) and the provision of formal psychological support (for patients). One construct ‘Guidelines and Evidence’ was not explicitly mentioned by patients and carers. However, a carer did comment that at time of referral to the hospice, the patient had been on two different does of co-codamol simultaneously ‘*You were on co-codamol, the 500/8 plus co-codamol 500/30’ (Patricia, carer)* which suggested to the researchers that the patient had been taking the medication in a way contrary to guidelines. Medications were then optimised by hospice staff. Four additional patient and carer-derived themes were identified: Carer Burden, Communication, Medicines management and Impact of COVID-19 (Fig. 1).

### Carer burden

Our HCP-based conceptual model identified a role for the carer in shaping symptom management experience in either a positive or negative way [1]. The patient and carer derived data presented here provides additional insight into their role and the activities required of them. Carer burden is a multifaceted experience, however our interview schedule specifically asked about symptom management experience.

The carer was sometimes responsible for raising concerns and initiating the referral for specialist palliative cares support *‘it was at some stage earlier in this year when I was a little anxious about your health and contacted the chemo wing at (hospital) and one of the nurses there thought it would be helpful to me and Patient to put us in touch with (the hospice) (Kathleen, carer).*

Carers were enmeshed into the disease and symptom experience of the patient, referring to ‘we’ when talking about the patient’s cancer treatment, pain and referral to hospice.

*Olivia (carer): Immune therapy we’d had a reaction to and we’d resolved the reaction but it concluded in stopping any treatment and we then went to a situation where we were not able to manage the pain from the cancer successfully and it was recommended by our* *oncologist that (the hospice) may have some expertise that we could….*

*Olivia (carer): Tap into…as I say that was a difficult decision for us to agree for Anthony to go into (the hospice).*

However, on occasion the insight from the carer was not acted upon leading to a delay in support for distressing symptoms ‘*I kept saying to people, he’s losing weight, he’s in pain and they just kept saying well he shouldn’t be in this amount of pain ‘cos of what his bloods are like. And I kept saying well what you’re saying he should be like, I can tell you he’s not like and we’re not ones to you know erm (he) isn’t one to be bothering the doctor.’ (Sandra, carer).*

Once the patient was receiving palliative care the carer took responsibility for obtaining and retaining knowledge either because the patient could not, due to memory problems from medication, or their condition, or they were not willing to do this for themselves.

*Martin (patient): ‘she knows better than me ‘cos I’m always, I’m not very good at remembering stuff’*

*Martin (patient): I’m not interested no I understand you do have a very important role and she’s taken the lead on it now, that’s definitely the case’*

And with another couple

*Terry (patient): Sorry I’ve got my wife at the side of me ‘cos she knows better than me ‘cos I’m always, I’m not very good at remembering stuff.*

*Stacey (carer): I’m usually present yeah, I’m usually around. I tend to be the one that asks more questions.*

However, in our interviews occasionally discordance between patient and carer opinion was seen with the carer rating the symptoms more troublesome than the patient’s recollection.

*Interviewer: So was it (the pain) stopping you doing any activities that you had been able to do?*

*Marti, (patient): Oh I see, not particularly no*

*Mary (carer): I would probably disagree with that sorry. I would say that Martin’s management of the pain and our management of the pain and everything was kind of a constant thing, that’s all we, you know if felt like we were talking about it all the time, his pain’.*

Despite an integral role in facilitating effective symptom management carers could feel unacknowledged, specifically by hospital staff. ‘*at the same time they’re telling me I’m not a carer and yet you know Wendy would be in a very sorry state if I wasn’t on the ball all the time’ (Patricia, carer). *Specialist palliative care staff were better at providing acknowledgement and consideration of individual capabilities.

*Patricia (carer): ‘So they understand that I’m not sort of hale and hearty and I’ve got my limitations….and it’s just lovely them knowing and actually accepting that I am caring for patient, we are doing the best that we can and that they are there for us.’. *This simple step of acknowledgement was appreciated and a factor in allowing the carer to continue to support the patient.

*Olivia (carer): ‘You know I do feel that it’s about me as well, it’s not just about Anthony which, it is really all about Anthony but you know it’s important that I continue with my wellbeing in order that I can support and look after him’ . *

### Communication

The impact of communication of effective symptom management occurred at different levels. As would be expected, communication needed to be tailored to the background, previous experience and outlook of the individual. In particular, we noted that a patient who had a healthcare background themselves welcomed more in-depth discussion and input into decision making.

*Andrew (patient): I’ve dealt with people with cancers and terminal illnesses. Yeah, I know about syringe drives and everything…The important thing is to be able to discuss it and with my knowledge of medication as well, I mean I can discuss it in depth.’ .*

Interestingly, this person also equated being admitted to the hospice with the use of a syringe driver and end of life, illustrating that regardless of the patient’s professional background, a thorough explanation without any assumptions on understanding would still be necessary. Andrew (patient): *‘I mean I could go into (the hospice) at any time knowing this but with my work record and everything else, I know what it all entails I mean I’d probably go in and they’d probably want to put me on a syringe drive with Oramorph and Midazolam and Betamethasone and everything else and I know that is the beginning of the end once you start on the syringe driver and everything because it just puts you to sleep and just makes you comfortable and you don’t really have no quality of life’ .*

Patients and carers valued being able to get in contact with someone when difficulties arose. Kathleen (carer):*‘Ease of communication is important to us so it’s easy to get in touch with somebody’ .*

For some people, at the earlier stages after referral to the palliative care team, the only support that they required was just telephone contact.

*Kathleen (carer): ‘What we have at the moment is a phone number to call and another lady, a nurse who actually rings us probably about once a fortnight yeah to check if we have any anxieties, problems.’ .*

Palliative care professionals had a key role in mediating communication between patients and carers and other services. Kathleen (carer): *‘she said yes, do you think Harry would mind us contacting the GP you know and I said I’m sure he would, if I think it’s a good idea he’d go along with it so that’s what we did, she did, she contacted our GP which meant that we got a telephone appointment and something happened very quickly’ .*

This extended to explaining the purpose and results of tests such as X-rays.

*Stacey (carer): Yeah he went when he was admitted he went for an Xray and that was the hospice, it was (clinical nurse specialist) that had organised that. We didn’t really know what was happening in the hospital but we came home again and he didn’t really know why he’d had the Xray or anything.*

*So when he spoke to the nurse at (the hospice), she sort of went through it all with him and talked him through it and that was really informative and helpful*

There was a feeling that communication was better in specialist palliative care compared to the general National Health Service (NHS).

*Olivia (carer): ‘There is an awful lot to be learned from the NHS about liaising and communications they could learn an awful lot from the way that the palliative care is operating and running’.*

The carer also became an advocate for the patient’s needs and relaying information about symptoms and concerns to the healthcare professionals which the patient may not have themselves. Andrew (patient): ‘*I mean she (partner) tells (hospice nurse) things that I don’t’ cos‘ I mean I sometimes bottle quite a few things up and don’t say nothing but (partner) notices these things and then she will tell (hospice nurse) about them’.*

This was also seen during a research interview, where the patient was willing for the carer to ‘tell the story’ on their behalf.

*Mary (carer): Sorry I’m doing all the talking.*

*Martin (patient): Well no you need to because I’m useless.*

We identified that patients had unmet needs in communicating about their condition *‘ Yeah, erm, again it’s, people are very reticent to use the word cancer. So they balk at saying the word’ (Wendy, patient) *and symptom experience with family and friends other than their regular carer.

*Wendy (patient): I don’t know where she’s (my sister) at in terms of knowing about my symptoms and about the treatment I’m having, well no I do tell her actually, it’s not that I don’t but she has very bad arthritis…so I don’t push that too much because I’m thinking she’s actually in as much pain as I might be.’*

This lack of communication could come from a position of wishing to protect the feelings of family members:

*Wendy (patient): ‘Oh it’s been very difficult with family. You don’t know how much you want to tell them and you don’t know how far down the line you are anyway. I think over the years, I’ve been protecting my family’ )*

Sometimes there were other important conversations that had not been held with family members.

*Martin (patient): ‘I suppose my point in bringing up was because they’re particularly good kids and they are particularly, although I wouldn’t like them to hear me say it but they are, very good’ . *

### The work of medicines management

Medicines management was a time consuming and complex task, even for carers who has a background working in healthcare.

*Sandra (carer): ‘I’m having to ring back my fourth phone call today to see is it a week off or have they forgotten to give him it. The communication isn’t great and I kind of think you know I’m kind of used to the NHS I’m, I know to ring and that sort of thing but I do think, I think if someone isn’t, got a health background or that sort of background there’s a lot of left to guesswork’ .*

Commonly, the responsibility of managing the medicines could be delegated to the carer due to the side effects of the medication on the patient’s memory. It was felt that the patient would not have been able to manage by themselves. Mary (carer): ‘*a lot of the medication has made him not so aware, maybe a little bit muddled at times and his memory’s not as good as it was….you know he does forget quite easily so I wouldn’t, I have to say I wouldn’t trust him with his medication at all.’.*

Carers took responsibility for ensuring medications were taken on time. As previously reported, this carer viewed this a joint endeavour with the patient.

*Patricia (carer): I wake (patient) at 9 o’clock and make sure that she has her Lansoprazole and that she has her 12 hourly Longtech tablet. I generally am doing everything and as I say, we put the injection in at lunchtime every day and at night I remind her, not that she doesn’t, she doesn’t really need reminding but at 9 o’clock, I say have you had your tablets?’ .*

The carer (who did not have a healthcare background) had developed an understanding of complex concepts such as the different modes of metabolism of medication for pain.

*Patricia (carer): ‘So she’s now on a different set of pain relief which, the morphine was better but not better for her. So the pain killing stuff that she’s on is processed through the liver rather than through the kidneys and the kidney function has stabilised.’ .*

### Impact of COVID-19

Interviewees were asked about whether COVID-19 had impacted upon their experience. It seemed that for this selected group of patients and carers the impact was minimal.

*Patricia (carer): ‘Can I just add that Covid seems to have, people have been complaining that this has stopped and that’s stopped whereas with Wendy her appointments, they’ve always wanted face to face and we’ve done phone appointments when it’s been appropriate and the care has been absolutely marvelous’.*

Availably of hospice staff sometimes filled the gap in other services.

*Kathleen (carer): ‘Because of lockdown and the virus and everything obviously all that (GP support) changed and you did start to feel a bit isolated and alone ‘cos you don’t always want to have to get in the car and drive to (hospital) for something if it’s not absolutely necessary and so therefore having someone else to talk to who knew more about things because obviously we’re learning as we go along Harry and I, it was very helpful’.*

Problems were attributed to the general NHS system rather than being COVID-19 specific.

*Sandra (carer): ‘I think as far as forthcoming information, I don’t think Covid has any bearing on that to be honest. You know, it just, I think it’s just an age-old problem in the NHS is communication.’ .*

## Discussion

The close alignment of this patient and carer data with our HCP-based conceptual model provides additional reinforcement of the importance of multidisciplinary working and continuity of care in shaping symptom management experience. Indeed, the ability to see preferred member of general practices staff was recently reported as a factor associated with satisfaction with ends of life care in England [3].

Palliative care takes a holistic view of the patient and carer, the concerns of both being intertwined and interdependent. The observation that carers and patients viewed themselves as a single unit and talked about ‘we’ when describing the experience of symptoms and service referral, aligns with the dimension of the carer ‘living in the patients world’ and living in ‘symbiosis’ recently described by Borelli et al [4] and in earlier qualitative work with advanced cancer patients [5]. Carer opinion can be a close but not always perfect proxy of patient voice, even in this small sample we observed some discordance between patient and carer perception of symptom burden. However, carers were vitally important for communication with healthcare providers, relaying concerns, managing medication and generally advocating for the patient when they were unable or willing to do so. In the UK in 2022, the number of people living alone was 8.3 million. Since 2020, the number of people over 65 years old living alone has also increased [6]. Household composition is not a general indicator of wider social support networks, but these data do suggest that there could be a considerable number of people with palliative care needs without live-in carer support. This raises the questions of whether the experience of those living without a supportive carer can be equitable and how services might better facilitate this.

Home-based palliative care is thought to reduce symptom burden for patients with cancer [7]. To enable this, it is therefore vital that carers are adequately supported. Carer burden is a multifaceted experience, however our interview schedule specifically asked about symptom management experience. In agreement with the term ‘role strain’ in the review by Choi and Seo [8] we saw carers involvement in symptom management and in mediating communication between the patient and healthcare providers. Additional aspects reported by Choi et Seo include physical symptoms of the carer, psychological distress, impaired social relationships, spiritual distress, financial crisis, disruption of daily life and uncertainty [8] and these will not have all been probed by our interview topic guide.

Although in our original study HCPs talked about medicines from their perspective, the role of the carer was not discussed. Medicines management was an important way that carers facilitated effective symptom management but is a complex task. One carer commented: *‘I have to say that would be a nightmare if I wasn’t a nurse by background’*. Our data on the difficulties with medicine management are not novel and closely mirror the report of Pollock et al., [9]. Our findings echo and support their conclusions that managing medicine at home during end-of-life care could be improved by reducing the work of medicines management and improving co-ordination and communication in health care and we echo their calls for further research in the area.

We identified that patients and carers viewed mediating communication as an important role for healthcare professionals. This could be enabling communication between patients and carers and other healthcare professionals, for example arranging follow-up care or explaining information received. There was also a need for better communication between patients and their family members. As reviewed and synthesised by Murray et al., (2014) the importance of effective communication in palliative care has been long recognised [10]. In our study, an opportunity for HCPs to facilitate better communication about symptom experience between patients and their wider family was identified. Our previous survey of English hospices found that healthcare professionals, particularly nurses and allied health professionals felt that they needed more training in basic and advanced communication skills [11]. Having relevant experience and if the appropriate training was provided, staff may be well placed to support patients with developing an approach to these potentially difficult conversations. Participants were offered a choice of joint or individual interviews, but most chose to be interviewed as a dyad. It is possible that being interviewed as a pair may have altered the information disclosed. Although the aim was to discuss factors that impacted upon effective symptom management, discussions at times deviated to a more general appraisal of a participant’s experiences and all data collected may not be relevant to the research question.

When data was collected that lead to the development of the HCP-based model of effective symptom management (May to November 2019) a global pandemic was unforeseen. At the time of the patient and carer interview described here (October to December 2020), COVID-19 restrictions were in place in the UK. The patients and carers we interviewed were already receiving specialist palliative care support as outpatients. For these individuals it appeared that the impact of COVID-19 pandemic had had minimal impact on their care. The availability and reassurance of telephone support from hospice staff seemed in part to ameliorate the reduced support available from other services such as GPs. This contrasts sharply with the negative impact of COVID-19 on the experience of patients and carers in the more immediate end of life phase [12], receiving oncology care [13] or with cancer more generally [14]. Selection bias is likely as patients and carers with the capacity and willingness to participate in our research study possibly reflect those where the illness is in a more stable phase and immediate needs were being met. Indeed, participants talked about difficulties before referral to specialist palliative care and with other services but were overwhelmingly positive about the support currently being provided by the hospice.

### Limitations

Due to the constraints of conducting a research study during the COVID-19 lockdown, more purposive sampling was not possible, this led to a lack of diversity in our sample. All participants identified themselves as of white British or white Scottish ethnicity which potentially means issues related to diverse ethnicities were not captured. All the patients who participated (and the non-participating patient whose carer was interviewed) lived with another person and had carer/family support. The experience of those managing their symptoms in isolation was therefore not captured. All participants were currently accessing support from a single hospice, the experience of those not yet receiving specialist support or receiving support from a different organisation may differ. The sample were diverse in age and included males and females, but all carers were female. Demographic information was not collected on socioeconomic background. COVID-19 restrictions necessitated the use of telephone interviews which may have lost subtle communications cues such as body language or conversely may have facilitated candid description. The transcripts do suggest that participants felt comfortable to tell their experience and they mostly spoke freely with limited prompting. One participant mentioned that he found it very difficult to leave the house, and therefore a telephone interview might have facilitated his inclusion. In some interviews more data was derived from the opinion of the carer than the patient, with the pair agreeing that the carer took responsibility for many tasks involved in managing the condition. We cannot be certain that carer interpretation accurately matches patient experience for all symptoms [15].

## Conclusions

We set out to answer the question; does a healthcare professional data derived model represent the experience of patients and carers of people with advanced cancer? Overall, the answer was yes, as our healthcare professional based conceptual model of effective symptom management aligned well with the experience of patients with advanced cancer and their carers. Domains that did not align were those specifically related to professionals; experience (of staff), training (of staff), guidelines and evidence, psychological support (for staff) and the provision of formal psychological support (for patients), a resource patients and carers might be unaware of. Additional domains of carer burden, communication, medicine management and the impact of COVID-19 were identified. We make four recommendations arising from this research.

1. Routine appraisal and acknowledgement of carer burden, medicine management tasks and previous experience in healthcare roles.
2. Increased access to communication skills training for staff caring for palliative care patients and their families.
3. Review of patient communication needs with support provided where needed.
4. Further research into the symptom management experience of those living alone and exploration of how these people can be better supported.

## Supplementary Information

Supplementary Material 1

Supplementary Material 2

Supplementary Material 3

Supplementary Material 4

Supplementary Material 5

## Data Availability

Original recordings generated and analysed during the current study are not publicly available due to protection of confidentiality. Anonymised transcripts with identifiable information removed may be available from the corresponding author on reasonable request.

## Abbreviations

COVID-19: Coronavirus disease 2019
HCP: Healthcare professional
NHS: National Health Service
UK: United Kingdom
