# Correction: Updating a conceptual model of effective symptom management in palliative care to include patient and carer perspective: a qualitative study

**DOI:** 10.1186/s12904-024-01571-8

**Published:** 2024-10-04

**Authors:** Emma J. Chapman, Carole A. Paley, Simon Pini, Lucy E. Ziegler

**Affiliations:** 1 Academic Unit of Palliative Care, Worsley Building, University of Leeds, Clarendon Way, LS2 9NL UK; 2 Division of Psychological and Social Medicine, Worsley Building, University of Leeds, Clarendon Way, LS2 9NL UK

**Correction: BMC Palliat Care 23**,** 208 (2024)**

10.1186/s12904-024-01544-x

Following publication of the original article [1], The author requested to changed the word from “sponsor” to “funder”.

On line 500 it reads.

‘The **sponsor** had no role in study design or the collection, analysis and interpretation of data; in the writing of the report; and in the decision to submit the article for publication.’.

This changes the correct meaning which should be.

‘The **funder** had no role in study design or the collection, analysis and interpretation of data; in the writing of the report; and in the decision to submit the article for publication.’.

The original article has been updated.
