# Supplementary material for: Updating a conceptual model of effective symptom management in palliative care to include patient and carer perspective: a qualitative study
Source: BMC Palliat Care. 2024 Aug 19;23:208. doi: 10.1186/s12904-024-01544-x (PMC11331639; doi:10.1186/s12904-024-01544-x)
Supplement: Supplementary file 1 — Supplementary Material 1 [file 12904_2024_1544_MOESM1_ESM.pdf]

Hello, my name is (insert name), I am one of the researchers on the RESOLVE research team.

We are here to talk about your experience of how your/your loved one's symptoms are managed by hospice staff.

Before we start the interview/workshop, I would like to make sure that you understand what we are going to do, how we will use the data we collect and give your consent to take part.

First of all, do you have any questions about the interview/workshop today?

I am going to ask you to confirm you name and the date today and then ask you to listen carefully to 12 statements that are written on the consent form and answer "Yes" or "No" to each one.

Note - (Where there are multiple participants, each will be asked in turn about each statement).

If you wish, you can put your initial next to each one as we go though as your own record. If you do not understand anything, please let me know.

The questions and your answers are being audio recorded as a record of your consent to take part in the research.

Please can you confirm your name and the date today.

1. Have you read and understood the information sheet dated 5/10/22 explaining the research project and had the opportunity to ask questions about the project?

2. Do you understand that participation is voluntary and that you are free to withdraw at any time during the interview/ focus group without giving any reason?

3. Do you understand any information you provide is for research purposes only.

4. Do you understand anonymised quotations (your words) from the focus group or interview may be used in publication of the results of the study.

5. Do you understand that the focus group or interview will be audio recorded and that the recording will not be shared with anyone outside of the research team

6. Do you understand that data collected during the study may be looked at by individuals from regulatory authorities or from the University of Leeds. Where it is relevant to you taking part in this research, do you give permission for these individuals to have access to your records.

7. Do you agree for the anonymised data collected from you to be stored and used in relevant future research?

8. Are you willing to be contacted to be invited to take part in future research?

9. Would you like to find out about the results of the study in the future and are happy for your contact details to be used for this purpose?

10. If in the unlikely event that during the research, I disclosure issues that the researchers feel may need urgent attention from my clinical team, do you give permission for the researcher to contact the research nurse at my hospice and/or staff involved in my care.

11. Do you understand that you have until 2 weeks following an interview to withdraw from the study. You can do so by contacting the researchers. Once the data has been fully anonymised it will not be possible to identify individuals and withdrawal will not be possible.

(Do you understand that it may not be possible to remove your participation from a focus group as it is a group activity?) If relevant.

12. Do you agree to take part in the study?

Thank you
