# Supplementary material for: Updating a conceptual model of effective symptom management in palliative care to include patient and carer perspective: a qualitative study
Source: BMC Palliat Care. 2024 Aug 19;23:208. doi: 10.1186/s12904-024-01544-x (PMC11331639; doi:10.1186/s12904-024-01544-x)
Supplement: Supplementary file 2 — Supplementary Material 2 [file 12904_2024_1544_MOESM2_ESM.pdf]

UNIVERSITY OF LEEDS RESOLVE Resolving Symptoms in Cancer

`

Participant information sheet

Study title: Effective management of symptoms in palliative care

We would like to invite you to take part in our research study. To help you decide whether you would like to take part or not, it is important you understand why the research is being done and what it will involve. Please carefully read the information below and ask questions if you would like any more information. If you are happy to participate you will be asked to sign a consent form.

We will only collect information that we need for the study. Everyone involved in this study will keep your data secure by following the University of Leeds privacy rules. At the end of the study, we will save some of the data for future research. We will make sure no-one can work out who you are from the reports we write. The information sheet below tells you more about this.

What is the purpose of the study?

The study is part of a research program aimed at improving the care of patients with advanced diseases.

We have already spoken to over 60 healthcare professionals who work in hospices about what that they think impacts upon whether management of pain, breathlessness and fatigue is successful.

- The purpose of this study is to now understand what patients and people important to patients (e.g family, friends, partners) think about these findings.

- The research team would also like to ask you about what aspects of care you think are most important in successfully managing symptoms and what you think would be most useful.

- We will put together what healthcare staff and patients and carers have told us and use this information to develop some new approaches to symptom management which we call interventions.

By doing this research, we hope to develop and design better approaches to the management of symptoms in the future.

`

Who is doing the study?

The research program, funded by Yorkshire Cancer Research (L412), is led by Professor Lucy Ziegler at the Academic Unit of Palliative Care, School of Medicine at the University of Leeds. Dr Emma Chapman and Dr Carole Paley are researchers from the University of Leeds.

Who should take part?

We would like to speak with patients who are cared for by the hospice (as inpatient, day services or in the community) and also people important to them such as family members, friends or partners.

Do I have to take part?

No, it is entirely up to you to decide whether or not to take part. Even if you agree to take part you can change your mind and withdraw from the study without giving any reason.

What happens if I change my mind?

You can change your mind and stop the interview or focus group at any time without giving a reason. If you would like to withdraw from the study, this will be possible up to 2 weeks after your interview or focus group. Please contact the researchers.

What will be involved if you take part in this study?

This study involves attending either a group meeting "focus group" with 2 or 3 other hospice patients or an individual interview. Focus groups or interviews will last less than 60 minutes. Focus groups will be held in a private room at the hospice. Individual interviews might be held at the hospice or other suitable place of your choice. The researcher can come to your home if this is preferred. If necessary, we may also carry out individual interviews over the telephone or using Microsoft Teams.

UNIVERSITY OF LEEDS RESOLVE Resolving Symptoms in Cancer

If you agree to take part, you will be contacted by the researcher and on the arranged date, the researcher will obtain your consent and then conduct the focus group or interview. You will be given an opportunity to ask any questions. The interviews will be recorded, using an encrypted audio recorder. Videos will not be recorded.

What are the possible benefits of taking part?

There is no compensation or direct benefit for participating in this study. Your participation will be a valuable and much appreciated addition to our research and we hope what we learn from this project will help inform on the future healthcare of many patients in the future. Taking part will give the opportunity for your views and experiences to be heard.

What are the possible risks of taking part?

It is not considered that there are any risks of being involved in this study. If you take part in a focus group with other people we will ask that you all respect the confidentiality of each other. However, we cannot guarantee this on behalf of the other participants. If during your interview or focus group you tell us about something that we feel needs urgent attention, we will talk to you about this and contact the hospice clinical team so that they may help with the problem.

How will we use information about you?

The University of Leeds is the sponsor for this study based in the United Kingdom. We will be using information from you in order to undertake this study and will act as the data controller for this study. This means that we are responsible for looking after your information and using it properly.

The information we will collect includes your name and contact details. This information will be used to do the research or to check your records to make sure that the research is being done properly. People who do not need to know who you are will not be able to see your name or contact details. You will be given a code number and no reference to personally identifiable data will be made. The University of Leeds will keep identifiable information about you for 5 years after the study has finished. Further relevant studies will be carried out in the next 5 years, and you can choose on the consent form if you would like us to contact you for another study.

UNIVERSITY OF LEEDS   RESOLVE Resolving Symptoms in Cancer

All data collected for this study will be anonymised and stored confidentially. Paper copies of your data will be kept in a locked filing cabinet in the researcher's office at the University of Leeds, UK. The interview recordings will be downloaded as soon as possible onto a password-protected computer and stored on a University of Leeds computer server. Once the recordings are uploaded to the secure server, they will be deleted from the audio recorder. Only researchers involved in this study will be able to identify you from your code number. This code number will be used when using any of your words (as illustrative quotations) in the report of findings. Your interview will be

transcribed by a professional transcriber and the transcripts stored securely on University password protected computers. We may use direct quotations from the interviews or focus groups, but individual participants will not be identified in any results arising from this study. Anonymised data may also be used in future research projects. This information will not identify you and will not be combined with other information in a way that could identify you. The information will only be used for the purpose of health and care research.

Your rights to access, change or move your information are limited, as we need to manage your information in specific ways in order for the research to be reliable and accurate. To safeguard your rights, we will use the minimum personally-identifiable information possible. The data handling procedures will be managed in accordance with the Data Protection Act 2018, the University of Leeds policies (The University data protection officer can be contacted at dpo@leeds.ac.uk, Research Participant Privacy Notice https://dataprotection.leeds.ac.uk/wp-content/uploads/sites/48/2019/02/Research-Privacy-Notice.pdf), and the EU General Data Protection Regulation (GDPR).

What will happen to the results of the research?

Your personal details will remain strictly confidential. Research findings made available in any reports or publications will not include information that can directly identify you without your specific consent. Research findings will be widely shared for example by publication in journals and presentations at medical and research conferences. Please initial the box on the consent form if you would like to read a summary of the research findings.

Who has reviewed this study?

The North of Scotland (1) Research Ethics Committee, which has responsibility for scrutinising all proposals for medical research on humans, has examined the proposal

and has raised no objections from the point of view of research ethics. It is a requirement that your study data in this research be made available for scrutiny by monitors from the University of Leeds, whose role is to check that research is properly conducted and the interests of those taking part are adequately protected.

What if I have concerns about this study?

If you have any concerns or for independent advice about taking part in this study, you can contact the Head of Research integrity and Governance, Clare Skinner Address: Faculty of Medicine & Health, Room 9.29, Worsley Building, University of Leeds, Leeds, LS2 9NL. Email: governance-ethics@leeds.ac.uk; Tel: 0113 343 4897

What next?

If you are interested in taking part, please complete the permission to contact form or contact the researchers using the contact details provided below.

Thank you for reading this information sheet and considering taking part in this study.

If you need any further information or have any questions or concerns about the study please get in touch with us.

Contact details:

Dr Emma Chapman

Tel: 0 113 343 0863

Email: e.j.chapman@leeds.ac.uk

Address: Academic Unit of Palliative Care, Leeds Institute of Health Sciences (LIHS), University of Leeds, Room 10.39, Level 10, Worsley Building, Clarendon Way, Leeds, LS2 9NL.
