# Supplementary material for: Updating a conceptual model of effective symptom management in palliative care to include patient and carer perspective: a qualitative study
Source: BMC Palliat Care. 2024 Aug 19;23:208. doi: 10.1186/s12904-024-01544-x (PMC11331639; doi:10.1186/s12904-024-01544-x)
Supplement: Supplementary file 3 — Supplementary Material 3 [file 12904_2024_1544_MOESM3_ESM.pdf]

UNIVERSITY OF LEEDS

RESOLVE
Resolving Symptoms in Cancer

Patient or Carer Consent Form

	Please initial box below
1. I have read the study information sheet dated xx/xx/xx and have had the opportunity to ask questions	
2. I understand that participation is voluntary and that I am free to withdraw at any time during the interview/ focus group without giving any reason	
3. I understand any information I provide is for research purposes only	
4. I understand anonymised quotations (my words) from the focus group or interview may be used in publication of the results of this study	
5. I understand the focus group or interview will be audio recorded and that the recording will not be shared with anyone outside of the research team	
6. I understand data collected during the study may be looked at by individuals from regulatory authorities or from the University of Leeds. Where it is relevant to my taking part in this research, I give permission for these individuals to have access to my records	
7. I agree for anonymised data collected from me to be stored and used in relevant future research	
8. I am willing to be contacted to be invited to take part in future research (please circle answer)	Yes / No
9. I would like to find out about the results of the study in the future and I am happy for my contact details to be used for this purpose (please circle answer)	Yes / No
10. In the unlikely event that during the research, I disclose issues that the researchers feel may need urgent attention from my clinical team, I give permission for the researcher to contact the research nurse at my hospice and/or staff involved in my care.	
11. I understand that I have until 2 weeks following my interview/focus group to withdraw from the study. I can do so by contacting the researchers. Once the data has been fully anonymised it will not be possible to identify individuals and withdrawal will not be possible.	
12. I agree to take part in this study	Yes / No

Participant number……………………….

Participant's Name (in block letters) ………………………………………………

Signature.. Date**:** ..../......./.......

Researcher's Name (in block letters)………………………………………………

Researcher's Signature……………………………………… Date: …../…../……..

A copy of the consent form will be retained and stored securely by the research team at the University of Leeds
