# Supplementary material for: Updating a conceptual model of effective symptom management in palliative care to include patient and carer perspective: a qualitative study
Source: BMC Palliat Care. 2024 Aug 19;23:208. doi: 10.1186/s12904-024-01544-x (PMC11331639; doi:10.1186/s12904-024-01544-x)
Supplement: Supplementary file 4 — Supplementary Material 4 [file 12904_2024_1544_MOESM4_ESM.pdf]

Appendix 2 Interview schedule –patients
5/10/22 V1.2 IRAS Project ID 280269

| Theme | General Question | Prompt items |
|---|---|---|
| Introduction | Introduce Research – Looking at how to make it easier to manage symptoms in advanced cancer patients | • Introduce self<br>• Explain confidentiality, length of interview/group, nature of discussion<br>• What we are going to cover (some background, then 2 card sort tasks with examples from your own experience)<br>• Any questions<br>• Obtain consent<br>• Start recording<br>• Participants invited to introduce themselves |
| Patient background | When did you become involved with palliative care? | • What difficulties were you experiencing at the time?<br>• Can you give us some examples of what the palliative care staff have supported you with? |
| Card sort 1<br>How decisions are made | What are the most important things when you and your care team decide how to manage your symptoms? | Can you tell me a little more about how you and your care team make decisions about your care?<br>• Is there something we have not covered here?<br>• Can you give me an example of when you were particularly happy with the way a decision was made about your treatment or care?<br>• Can you tell me about a time when you were less happy with how a decision was made about your treatment or care? |
| Card sort 2<br>What factors influence the success of approaches to manage symptoms, side-effects and other problems | What are the most important things to you when receiving support for your symptoms? | Can you tell me a little more about how these things affect you?<br>• Is there something we have not covered here?<br>• Can you give me an example of something that has really helped you?<br>• Can you tell me about something that didn't work well for you? |
| Closing | Thank you for your participation | |
