# Supplementary material for: Updating a conceptual model of effective symptom management in palliative care to include patient and carer perspective: a qualitative study
Source: BMC Palliat Care. 2024 Aug 19;23:208. doi: 10.1186/s12904-024-01544-x (PMC11331639; doi:10.1186/s12904-024-01544-x)
Supplement: Supplementary file 5 — Supplementary Material 5 [file 12904_2024_1544_MOESM5_ESM.pdf]

Appendix 3.Interview schedule - carers
5/10/22 Version 1.2 IRAS Project ID 280269

Theme	General Question	Prompt items
Introduction	Introduce Research – Looking at how to make it easier to manage symptoms in advanced cancer patients	• Introduce self<br>• Explain confidentiality, length of interview/group, nature of discussion<br>• What we are going to cover (some background, then 2 card sort tasks with examples from your own experience)<br>• Any questions<br>• Obtain consent<br>• Start recording<br>• Participants invited to introduce themselves
Patient background	When did the patient become involved with palliative care?	• What difficulties were they experiencing at the time?<br>• Can you give us some examples of what the palliative care staff have supported them and you with?
Card sort 1<br>How decisions are made	What are the most important things when the care team decide how to manage the patient's symptoms?	Can you tell me a little more about how the care team make decisions about the patient's care?<br>• Is there something we have not covered here?<br>• Can you give me an example of when you were particularly happy with the way a decision was made about the treatment or care?<br>• Can you tell me about a time when you were less happy with how a decision was made about the treatment or care?
Card sort 2<br>What factors influence the success of approaches to manage symptoms, side-effects and other problems	What are the most important things when the patient is receiving support for their symptoms?	Can you tell me a little more about how these things affect the patient or you ?<br>• Is there something we have not covered here?<br>• Can you give me an example of something that has really helped the patient or you ?<br>• Can you tell me about something that didn't work well for the patient or you ?
Closing	Thank you for your participation	
